# Design of Non-Uniform Antenna Arrays for Improved Near-Field MultiFocusing

**DOI:** 10.3390/s19030645

**Published:** 2019-02-04

**Authors:** Rafael González-Ayestarán, Jana Álvarez, Fernando Las-Heras

**Affiliations:** Group of Signal Theory and Communications, Universidad de Oviedo; 33203 Gijón (Asturias), Spain; jalvarez@tsc.uniovi.es (J.Á.); flasheras@uniovi.es (F.L.-H.)

**Keywords:** antenna arrays, near-field focusing, optimization, pattern synthesis

## Abstract

An extended method for Near-Field Multifocusing on antenna arrays, including the optimization of the locations for the elements of the array, is proposed. Multifocusing is gaining attention in recent years due to the growth of applications such as Internet of Things, or 5G, where a wireless link between a number of sensors and devices must be established, and energy or interference must be managed efficiently. Multifocusing requirements may be addressed by optimizing the feeding weights that must be applied to the elements of an array, but the proposed methodology also optimizes their locations, increasing the degrees of freedom by allowing a non-uniform structure for the array, leading to more efficient structures or better compliance with the specifications. Some experiments are presented to validate the method, showing that it is able to determine the weights and mesh of the array to fulfill the requirements, both obtaining an arbitrary distribution of elements or following a predefined geometric model.

## 1. Introduction

Near-Field (NF) techniques [1,2,3,4] are gaining increasing relevance in recent years due to the growth of applications such as Internet of Things (IoT) or 5G mobile telephony that require establishing wireless links between sensors and devices typically located at short distances, in most cases into the Near-Field region of their antennas. Morever, efficient energy management is requested to avoid wasting energy in locations where no devices are present, as provided by techniques such as Wireless Power Transfer (WPT) or Wireless Power and Information Transfer (WPIT) [5,6]. Near-Field Focusing (NFF) has been shown to be extremely useful to achieve these objectives, as it is able to concentrate most of the radiated energy at an assigned position, the so-called focal point, where the device to be linked is located. However, conventional approaches to NFF are limited to only one focal point, while new applications usually involve multiple devices simultaneously.

Near-Field Multifocusing (NF-MF) on antenna arrays is a novel technique for concentrating the radiated field in certain assigned positions of the NF region of the antenna [7,8]. This technique arises as an alternative to traditional methods for NF focusing on one position, such as the Conjugate-Phase (CP) [9], which has been proven to be useful in applications such as RFID [1], medical systems [10] or weapon detection [11]. In the conventional CP approach, the phase of the weights applied at each element of an antenna array is modified to compensate the different distances to the focal point, so that all their individual contributions arrive in-phase at that focal point. It has been shown to be an excelent choice to solve one-spot problems, but it is not useful when multiple devices or sensors are involved. Other techniques have been proposed to overcome these limitations. They are related to the use of multiple-feed reflectarrays [12], leaky wave lenses [13,14,15], artificial neural networks [16], optimization approaches [7,17,18,19], or time-reversal techniques [8,18,19]. Among them, optimization approaches have been proven to be a flexible and powerful methodology, able to deal with general NFF problems, and also being the basis for the NF-MF framework. It allows focusing on multiple points simultaneously, assigning nulls or minimizing focal-shift effects in the peak positions [20] or spureous peaks caused by the shape of the radiated field in the rest of NF positions [17].

The NF-MF method [7] consists of the resolution of a Least Squares problem, based on the definition of a proper cost function where a range of allowed field levels is established for each location of the NF region. This function is minimized using the iterative Levenberg-Marquardt algorithm (LM) [21] as an optimization method, given its success in the resolution of nonlinear problems [7]. The definition of bounds or a mask for the radiated field in the cost function allows handling different types of requirements besides NF multifocusing, such as specification of nulls or focusing at arbitrary volumes or regions. Moreover, the flexibility of the method is also noted in the type of variables to obtain in the synthesis process, since either magnitudes and phases of the weights applied at each element of the array (magnitude-phase optimization, MP), or only their phases (phase-only synthesis, PO) can be configured.

On the other hand, there is a growing interest in designing non-uniform arrays, since the degrees of freedom achieved by considering the locations of the elements of the array may improve the antenna capabilities without increasing its cost or complexity [22,23]. Thus, a complete framework including location synthesis is proposed in this paper, allowing flexibility in the array unknowns: the previous MP and PO cases can be extended for location optimization, providing a complete Magnitude-Phase-Position or Phase-Position synthesis. Although the computational cost is increased with respect to the synthesis of uniform arrays (since the number of variables to obtain is higher), demanding specifications can still be handled. In addition, different meshes can be designed, providing more flexibility in the array structure. Linear distributions are disregarded since NF-MF requires asymmetrical 3D specifications, but the location of the elements can be follow a planar or non-uniform distribution, even acquiring three dimensional functions.

This paper is organized as follows; Section 2 reviews the mathematical model used for the NF distribution in order to include location optimization. Optimization is detailed in Section 3 according to the framework presented in [7]. Section 4 shows some validation results in order to compare location synthesis with the previous phase-only and magnitude-phase optimization, and to demonstrate the provided flexibility and accuracy. Finally some conclusions are outlined in Section 5.

## 2. Near Field Multifocusing on Antenna Arrays

**Figure 1:**
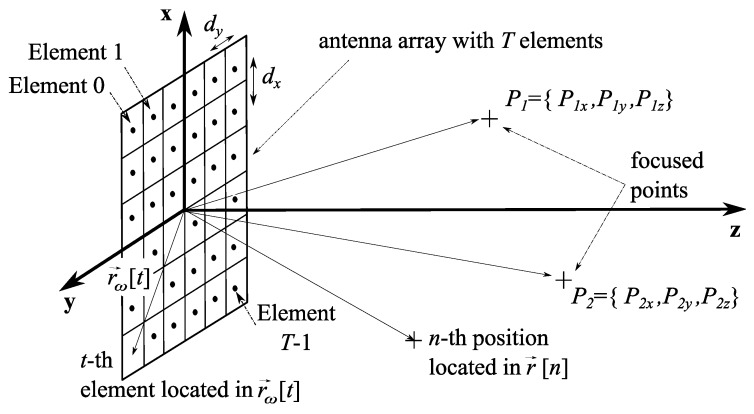
General scheme of a Near-Field (NF) Multifocusing problem, where the antenna array concentrates the radiated field at positions $P_{1},P_{2}$ (defined by their coordinates). Element and position vectors are also described.

**Figure 2:**
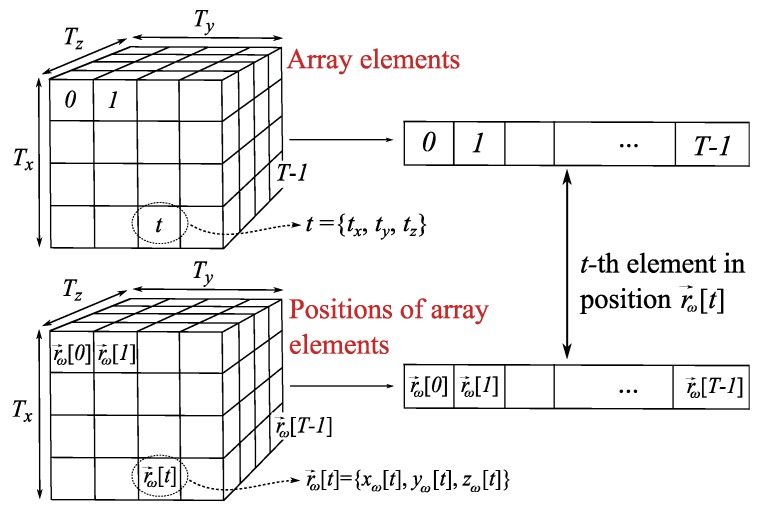
Relationship between elements and the positions where they are located by using index *t* and a vectorization process.

The NF-MF framework [7] uses a general model for an antenna array of $T=T_{x}\times T_{y}\times T_{z}$ elements, where $T_{x},T_{y},T_{z}$ are the number of elements in each direction/coordinate (x,y,z). The general scheme is represented in Figure 1, which is particularized for a planar antenna ($T=T_{x}\times T_{y}\times 1$) located at the plane z=0. The *t*-th element (t=0…T−1) is defined by its spatial coordinates as $t=\{t_{x},t_{y},t_{z}\}$, where $t_{x}=0\ldots T_{x}-1,t_{y}=0\ldots T_{y}-1,t_{z}=0\ldots T_{z}-1$ identify the element index in each direction. The total radiated field is expressed as the superposition of the *T* contributions as:

(1) $$\vec{E}\left(\vec{r}\right)=\sum_{t=0}^{T-1}\omega \left[t\right]{\vec{E}}_{t}\left(\vec{r}\right)$$

where $\vec{E}\left(\vec{r}\right)$ is the radiated field at position $\vec{r}$, ω[t] is the feeding weight to be applied at the *t*-th array element and ${\vec{E}}_{t}\left(\vec{r}\right)$ identifies the field radiated by the *t*-th element at $\vec{r}$. The sum in *t* used in (1) entails a vectorization process over the elements (and their locations), as it is explained in Figure 2, considering that the *t*-th element is located at ${\vec{r}}_{\omega }\left[t\right]=\left\{x_{\omega }\left[t\right],y_{\omega }\left[t\right],z_{\omega }\left[t\right]\right\}$, t=0…T−1. Note that expression (1) can be applied to arbitrary meshes, so spatial components $\left\{x_{\omega }\left[t\right],y_{\omega }\left[t\right],z_{\omega }\left[t\right]\right\}$ might be different for each element *t*.

**Figure 3:**
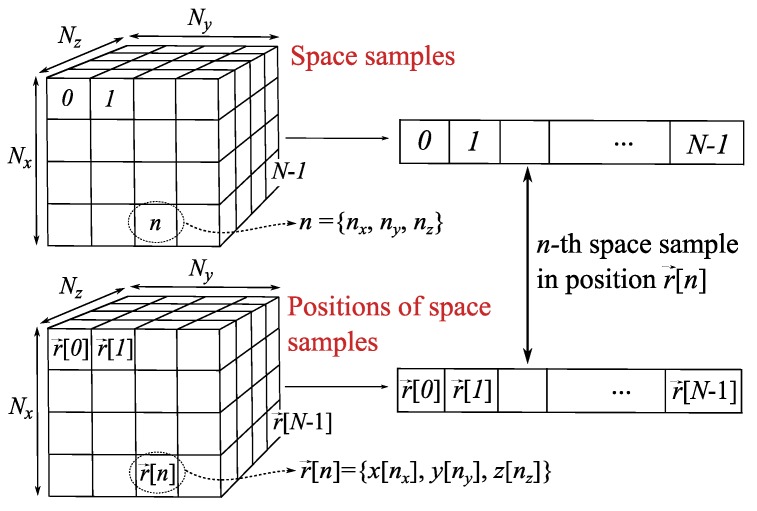
Relation between space samples and their locations by using index *n* after the discretization of $\vec{r}$ and vectorization.

In order to deal with the NF region surrounding the antenna array, $N=N_{x}\times N_{y}\times N_{z}$ sampled positions are taken into account to discretize $\vec{r}$, where $N_{x},N_{y},N_{z}$ define the number of considered positions in each direction. Considering $n_{x}=0\ldots N_{x}-1,n_{y}=0\ldots N_{y}-1,n_{z}=0\ldots N_{z}-1$ as the indexes to identify the number of position in each direction, the *n*-th sample (n=0…N−1) is defined by its spatial components $n=\{n_{x},n_{y},n_{z}\}$. Thus, $\vec{r}$ is discretized as $\vec{r}\left[n\right]=\left\{x\left[n_{x}\right],y\left[n_{y}\right],z\left[n_{z}\right]\right\}$ (see Figure 3), where a new vectorization process is carried out using index *n*. Note that uniform sampling is assumed, so spatial components only depend on their own index, i.e., $x\left[n\right]=x\left[n_{x}\right],y\left[n\right]=y\left[n_{y}\right],z\left[n\right]=z\left[n_{z}\right]$ (in contrast with element locations which can follow arbitrary meshes). Thus, the radiated field in (1) can be rewritten using *n* as:

(2) $$\vec{E}\left[n\right]=\sum_{t=0}^{T-1}\omega \left[t\right]{\vec{E}}_{t}\left[n\right]$$

where $\vec{E}\left[n\right]$ is the *n*-th sample of vector $\vec{E}={\left\{\vec{E}\left[0\right],\vec{E}\left[1\right],\ldots ,\vec{E}\left[N-1\right]\right\}}^{T}$, which contains the field values at the considered *N* positions. On the other hand, the radiation distribution of the *t*-th array element at the *n*-th point is given by:

(3) $${\vec{E}}_{t}\left[n\right]=\frac{1}{R[n,t]}e^{-j\beta R[n,t]}\vec{E_{o}}\left[n,t\right]$$

where β=2π/λ, with λ being the free-space wavelength, and $\vec{E_{o}}\left[n,t\right]$ is the *n*-th sample of the radiation pattern of the *t*-th element. It is interesting to notice that the FF pattern of the individual elements is used provided that the considered scenarios concern a radiative NF region for the array but a FF region for the individual elements is used provided that their size is much smaller, and hence their FF region is closer. Notice that the FF regions starts from the typical value of $R_{FF}=2D^{2}/\lambda$ [24], where *D* is the maximum dimension of the antenna, provided that $\beta \cdot R_{FF}>>1$. If resonant elements are considered, the last restriction applies for the element and we could establish a value of $R_{FF}>1.6\lambda$ for a good approximation of the eectric field radiated by the element using FF expression. From the point of view of the whole array, if a *T*-element array is considered, with an interelement distance of dλ, the FF region is located beyond $2{\left(T-1\right)}^{2}d^{2}\lambda$. For example, in a 16-element array with d=0.8, such an FF distance is 288λ. Hence, the region of interest for multifocusing purposes, for this array and neglecting coupling, could be considered at distances between 1.6λ and 288λ. If closer distances where involved, a NF formulation should be included for the elements of the array. In the following results, equal elements are being considered for simplicity, but this formulation can be modified for including coupling or different element radiation patterns [7]. Finally, R[n,t] accounts for the distance between the *t*-th element and the *n*-th spatial point:

(4) $$R\left[n,\;t\right]\;=\;\sqrt{\;{\left(z\left[n_{z}\right]\;-\;z_{\omega }\left[t\right]\right)}^{2}\;+\;{\left(y\left[n_{y}\right]\;-\;y_{\omega }\left[t\right]\right)}^{2}\;+\;{\left(x\left[n_{x}\right]\;-\;x_{\omega }\left[t\right]\right)}^{2}\;}\;$$

Equations (2) and (3) may be replaced by more complete model of the radiating system if required. For example, in [25] or in [16] both the radiation pattern of each element of the array and a matrix accounting for the coupling effects between elements has been considered. It is straightforward that a more complete model will lead to more accurate results, but also to slower computation. In a problem where electromagnetic accuracy is of great importance, the proposed equations might be replaced by a full-wave analysis tool (see [26] for detailed information) in exchange of computational cost, which may become unacceptable for many applications.

## 3. NF-MF Framework Including Position Optimization

### 3.1. Cost Function Definition

The objective of the NF-MF approach is to find the proper parameters of the array so that the radiated field in (2) is concentrated at certain assigned locations and reduced at any other position in the NF region [7]. For this purpose, the method is based on minimizing a proper cost function, *F*, which considers a range of values allowed for the field at each position *n*:

(5) $$\begin{array}{cc} \;F & \;=\;\sum_{n=0}^{N-1}\;C{\left[n\right]}^{2}\;\left[\;\left(G_{M}^{2}\left[n\right]\;-\;{\bar{|\vec{E}\left[n\right]|}}^{2}\right)\;\;\left(G_{m}^{2}\left[n\right]\;-\;{\bar{|\vec{E}\left[n\right]|}}^{2}\right)\;\;+\;\right.\;\\ & \;+\;{\left.\left|G_{M}^{2}\left[n\right]\;-\;{\bar{|\vec{E}\left[n\right]|}}^{2}\right|\;\;\left|G_{m}^{2}\left[n\right]\;-\;{\bar{|\vec{E}\left[n\right]|}}^{2}\right|\right]}^{2}\;\;=\;\sum_{n=0}^{N-1}\;F_{c}^{2}\left[n\right] \end{array}$$

where $G_{M}\left[n\right],G_{m}\left[n\right]$ are the maximum and minimum values allowed for the field radiated at the *n*-th position, respectively and the overline stands for normalization, forcing $0\leq G_{M}\left[n\right],G_{m}\left[n\right]\leq 1$; $C\left[n\right]\in {ℜ}^{+}$ is introduced to emphasize the error at the *n*-th position, being higher in those critical positions where the error must be minimum.

$F_{c}\left[n\right]$ represents the error for the *n*-th position, so $F_{c}\left[n\right]=0$ in the positions where the radiated field is in-bounds; thus, these addends can be rewritten as an equivalent least squares (LS) problem:

(6) $$\;\;F_{c}^{2}\left[n\right]\;=\;\left\{\begin{array}{c} 0,\;if\;G_{m}^{2}\left[n\right]\;\leq \;{\bar{|\vec{E}\left[n\right]|}}^{2}\;\leq \;G_{M}^{2}\left[n\right]\\ 4C^{2}\left[n\right]\;\;{\left[\;\left(\;G_{M}^{2}\left[n\right]\;-\;{\bar{|\vec{E}\left[n\right]|}}^{2}\;\right)\;\;\;\left(\;G_{m}^{2}\left[n\right]\;-\;{\bar{|\vec{E}\left[n\right]|}}^{2}\;\right)\;\;\right]}^{2}\;\;=\;{\left(y\left[n\right]\;-\;f_{c}\left[n,\alpha \right]\right)}^{2}\;,\mathrm{otherwise} \end{array}\right.$$

where y[n] represents the values to be approximated by the function $f_{c}\left[n,\alpha \right]$ by calculating vector α:

(7) $$f_{c}\left[n,\;\alpha \right]\;=2C\left[n\right]{\bar{|\vec{E}\left[n\right]|}}^{2}\;\left(G_{M}^{2}\left[n\right]\;+\;G_{m}^{2}\left[n\right]\;-\;{\bar{|\vec{E}\left[n\right]|}}^{2}\right)\;$$

(8) $$y\left[n\right]=2C\left[n\right]G_{M}^{2}\left[n\right]G_{m}^{2}\left[n\right]$$

In this problem, α contains the unknowns of the synthesis problem, i.e., the feeding weights and positions of elements, which define the radiated field $\vec{E}\left[n\right]$ and therefore $f_{c}\left[n,\alpha \right]$.

The minimization of (6) is carried out using the iterative Levenberg-Marquardt (LM) algorithm [21], found useful in [7] for the resolution of the LS problem in (6).

One of the benefits of using the proposed vectorization of the scenario (represented in Figure 2 and Figure 3), and especially the discretization of the NF region, is given by the reduced computation time required using (2) with respect to more sophisticated full-wave solvers when performing analysis. Optimization algorithms require the calculation of the cost function corresponding to the actual solution at each iteration, what requires the analysis of the antenna under study. In a simple optimization scheme performing only one analysis per iteration, the total computation time required to complete the minimization of the cost function associated to a synthesis problem is directly related to the time required for such analysis. If a simple expression such as (2) may be used, the total computation time may be drastically reduced. For example, a scenario with a 12 × 12 patch-element array, with interelement distance of 0.7λ (being λ the free-space wavelength), is considered. The NF region is limited to x∈[−10λ,10λ], y∈[−10λ,10λ], z∈[0,20λ], with space samples separated 0.5λ. This sampling represents 68,921 space samples, related to 256 array elements through Equation (2). The analysis to calculated the field radiated by the array takes a computation time of 0.019 s using the proposed vectorization (using Matlab R2018b on a computer with an Intel Core i5-7500 CPU at 3.4 GHz, 8 GB RAM, and Windows 10), while the same analysis, involving typically over 1 million unknowns, takes more than 20 h using a commercial solver (Ansys HFSS) on a workstation with 16 cores and 128 GB RAM (it cannot be addressed in the same computer). A complete optimization for a multifocusing problem requires repeating the analysis at each iteration, what means that using a non-vectorized method might overflow many applications. Instead, the use of the proposed vectorization reduces the size of the problem allowing the use of a conventional PC.

### 3.2. Magnitude-Phase-Position and Phase-Position Synthesis

The minimization of *F* using LM requires the setup of a Jacobian matrix, $J\in {ℜ}^{N\times A}$, where *A* is the length of α [7]. Its *n*-th row is filled with the partial derivatives of $F_{c}\left[n\right]$ with respect to the components of α; considering that only $f_{c}\left[n,\alpha \right]$ depends on α, each element follows:

(9) $$\frac{\partial F_{c}\left[n\right]}{\partial \alpha [a]}\;\;=\;\;\left\{\begin{array}{c} 0,if\;G_{m}^{2}\left[n\right]\leq {\bar{|\vec{E}\left[n\right]|}}^{2}\leq G_{M}^{2}\left[n\right]\\ \frac{-4C[n]}{max^{2}\left(\right|\vec{E}\left|\right)}\;\left\{\;\left[E_{R}\left[n\right]\frac{\partial E_{R}\left[n\right]}{\partial \alpha [a]}\;+\;E_{I}\left[n\right]\frac{\partial E_{I}\left[n\right]}{\partial \alpha [a]}\;\right]\;\left[\;\left(G_{M}^{2}\left[n\right]\;+\;G_{m}^{2}\left[n\right]\right)\;-\;2{\bar{|\vec{E}\left[n\right]|}}^{2}\;\right]\;\right\}\;,\mathrm{otherwhise} \end{array}\right.$$

where a=0…A−1 and $E_{R}\left[n\right],E_{I}\left[n\right]$ are the real and imaginary parts of the radiated field at the *n*-th position.

In order to build J, it is required the definition of α, which contains the array parameters to be obtained. The feeding weights in (2) modify the radiated field distribution, so the original NF-MF framework in [7] was focused on their synthesis: α was designed to contain either real and imaginary parts of the feeding weights, or only their phase terms, so a complete magnitude-phase (MP) optimization or a phase-only (PO) synthesis were the options purposed to fulfill the NF-MF specifications.

If element-location optimization is included, the position coordinates of the element locations can also be modified in order to obtain the desired NF distribution. The complete α for MP and PO cases is completed with the variables that define the positions where the elements must be located, providing a MPP (Magnitude-Phase-Position) or a PP (Phase-Position) synthesis:

(10) $$\alpha_{MPP}={\left[\omega_{R}^{T},\omega_{I}^{T},x_{\omega }^{T},y_{\omega }^{T},z_{\omega }^{T}\right]}^{T}$$

(11) $$\alpha_{PP}={\left[∠\omega^{T},x_{\omega }^{T},y_{\omega }^{T},z_{\omega }^{T}\right]}^{T}$$

where ω is the vector form of the feeding weights, i.e., $\omega ={\left[\omega \left[0\right],\omega \left[1\right],\ldots ,\omega \left[T-1\right]\right]}^{T}$, and $\omega_{R}=Re\left(\omega \right),\omega_{I}=Im\left(\omega \right)$ as in [7]; $x_{\omega },y_{\omega },z_{\omega }$ identify the vectors for the coordinates (or spatial components) of the elements location: $x_{\omega }={\left[x_{\omega }\left[0\right],x_{\omega }\left[1\right],\ldots ,x_{\omega }\left[T-1\right]\right]}^{T},y_{\omega }={\left[y_{\omega }\left[0\right],y_{\omega }\left[1\right],\ldots ,y_{\omega }\left[T-1\right]\right]}^{T},z_{\omega }={\left[z_{\omega }\left[0\right],z_{\omega }\left[1\right],\ldots ,z_{\omega }\left[T-1\right]\right]}^{T}$. Thus, the maximum number of unknowns is A=5T and A=4T for both cases respectively.

In order to complete (9), the partial derivatives with respect to the positions in α are defined as:

(12) $$\begin{array}{cc} \;\; & \;\;\frac{\partial E_{R}\left[n\right]}{\partial x_{\omega }\left[t\right]}\;=\;\frac{x\left[n_{x}\right]\;-\;x_{\omega }\left[t\right]}{R^{2}\left[n,t\right]}L_{1}\left[n,t\right]\\ \;\; & \;\;\frac{\partial E_{I}\left[n\right]}{\partial x_{\omega }\left[t\right]}\;=\;\frac{x\left[n_{x}\right]\;-\;x_{\omega }\left[t\right]}{R^{2}\left[n,t\right]}L_{2}\left[n,t\right]\\ \;\; & \;\;\frac{\partial E_{R}\left[n\right]}{\partial y_{\omega }\left[t\right]}\;=\;\frac{y\left[n_{y}\right]\;-\;y_{\omega }\left[t\right]}{R^{2}\left[n,t\right]}L_{1}\left[n,t\right]\\ \;\; & \;\;\frac{\partial E_{I}\left[n\right]}{\partial y_{\omega }\left[t\right]}\;=\;\frac{y\left[n_{y}\right]\;-\;y_{\omega }\left[t\right]}{R^{2}\left[n,t\right]}L_{2}\left[n,t\right]\\ \;\; & \;\;\frac{\partial E_{R}\left[n\right]}{\partial z_{\omega }\left[t\right]}\;=\;\frac{z\left[n_{z}\right]\;-\;z_{\omega }\left[t\right]}{R^{2}\left[n,t\right]}L_{1}\left[n,t\right]\\ \;\; & \;\;\frac{\partial E_{I}\left[n\right]}{\partial z_{\omega }\left[t\right]}\;=\;\frac{z\left[n_{z}\right]\;-\;z_{\omega }\left[t\right]}{R^{2}\left[n,t\right]}L_{2}\left[n,t\right] \end{array}$$

where:

(13) $$\begin{array}{c} L_{1}\left[n,t\right]=\left(S_{1}\left[n,t\right]E_{oR}\left[n,t\right]-S_{2}\left[n,t\right]E_{oI}\left[n,t\right]\right)\\ L_{2}\left[n,t\right]=\left(S_{2}\left[n,t\right]E_{oR}\left[n,t\right]+S_{1}\left[n,t\right]E_{oI}\left[n,t\right]\right)\\ S_{1}\left[n,t\right]=\left(\frac{B_{1}\left[n,t\right]}{R[n,t]}-\beta B_{2}\left[n,t\right]\right)\\ S_{2}\left[n,t\right]=\left(\frac{B_{2}\left[n,t\right]}{R[n,t]}+\beta B_{1}\left[n,t\right]\right)\\ B_{1}\left[n,t\right]=\omega_{R}\left[t\right]cos\left(\beta R\left[n,t\right]\right)+\omega_{I}\left[t\right]sin\left(\beta R\left[n,t\right]\right)\\ B_{2}\left[n,t\right]=\omega_{I}\left[t\right]cos\left(\beta R\left[n,t\right]\right)-\omega_{R}\left[t\right]sin\left(\beta R\left[n,t\right]\right) \end{array}$$

being $E_{oR}\left[n,t\right],E_{oI}\left[n,t\right]$ the real and imaginary parts of $E_{o}\left[n,t\right]$.

### 3.3. Selection of Array Parameters to be Synthesized

Given a predefined value of *T*, vector α (in both versions $\alpha_{MPP}$ and $\alpha_{PP}$) in (10) and (11) represents all the possible array parameters that can be optimized to comply with the radiation requirements, including the three components of the location of each element of the array. However, this entire optimization does not present a real advantage, taking into account fabrication issues or temporal costs in the iterative optimization method, especially if all three components of the locations are optimized, what could lead to an unrealizable array. Moreover, certain limits in the allowed positions must be established in order to consider the size of the elements and to avoid their stacking, or certain geometries may be imposed if the array is required to be non-planar (such as a paraboloid). Moreover, element location is also related to coupling effects between the elements of the array. The closer the elements are, more mutual coupling may affect the system. For this reason, certain additional constraints may be included in the optimization, to establish a lower limit of interelement distance. If a formulation accounting for coupling effects is used, such as the above mentioned in [16,25], the optimization scheme will also take them into account and the constraints may be limited to those required for implementation issues.

**Figure 4:**
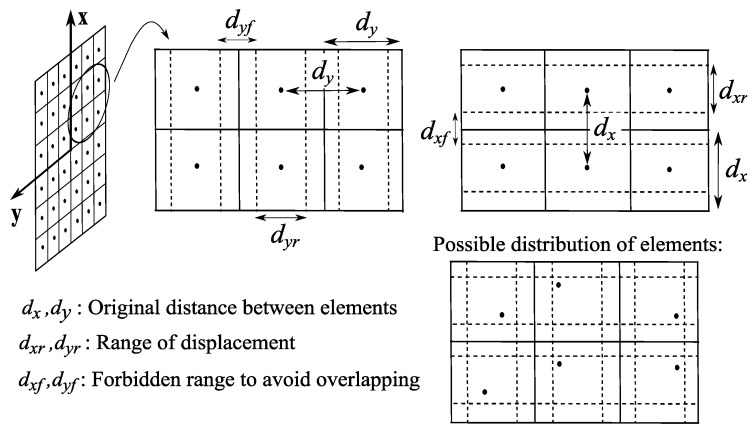
General scheme of range of positions allowed in the synthesis of a planar array antenna. Elements are represented with black dots. A forbidden area is defined to avoid overlapping.

**Figure 5:**
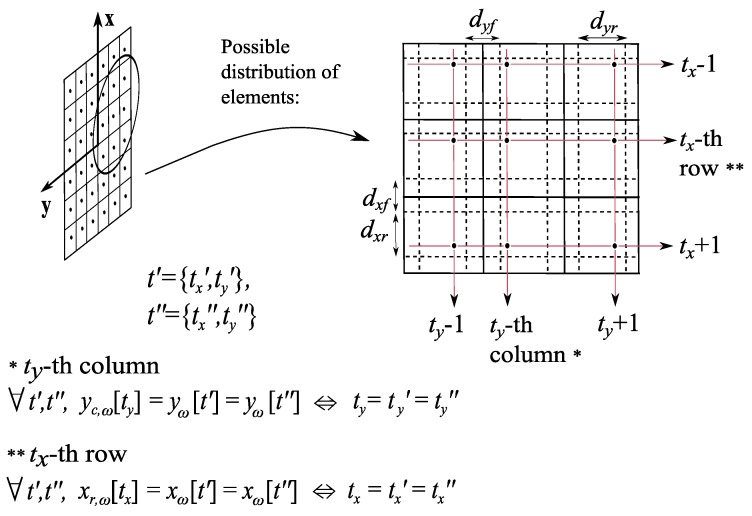
General scheme of range of positions allowed in the synthesis of rows and columns of a planar array antenna (elements are represented with black dots).

**Figure 6:**
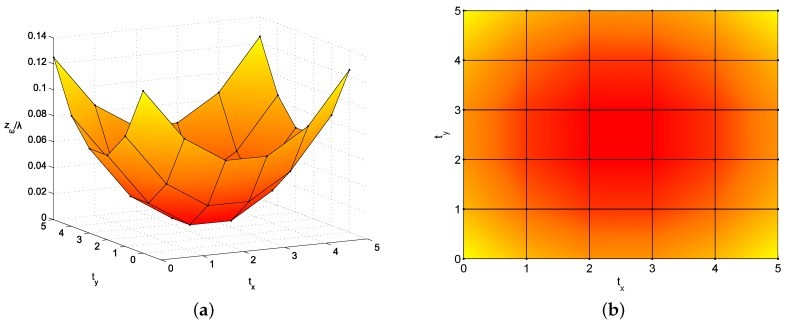
Example of mesh for a 6×6 antenna array, where elements follow a paraboloid function (a=b=5); component $z_{\omega }$ is modified (**a**) but $\left\{x_{\omega },y_{\omega }\right\}$ are fixed, with an uniform mesh (**b**). Elements are located in the vertices.

As the definition of the vector α allows flexibility in the kind of parameters to include, three different configurations are remarked:

- Planar array antennas with irregular mesh. If planar array antennas are considered, the optimization of positions can be limited to $\left\{x_{\omega },y_{\omega }\right\}$, avoiding the modification of coordinates $z_{\omega }$; thus, $\alpha_{MPP}={\left[\omega_{R}^{T},\omega_{I}^{T},x_{\omega }^{T},y_{\omega }^{T}\right]}^{T}$ and $\alpha_{PP}={\left[∠\omega^{T},x_{\omega }^{T},y_{\omega }^{T}\right]}^{T}$ (4T and 3T unknowns, respectively). A possible scheme which represents the limits in the allowed locations of the elements in the plane z=0 is represented in Figure 4. Note the non-uniform mesh allowed in both directions {x,y}.
- Planar array antennas keeping the distribution in rows and columns. In order to simplify the previous mesh, NF-MF algorithm may be modified to optimize positions maintaining the original distribution in rows and columns (i.e., only the location of each row and column is to be optimized), as it is depicted in Figure 5. This option highly reduces temporal costs, since the number of variables considered in the position synthesis is lower (only the number of rows and columns, i.e., $T_{x}+T_{y}$). Moreover, the fabrication issues are simplified with respect to the previous case; in contrast, the NF performance of the resulting array may decrease, since the number of degrees of freedom in the solutions is reduced, and hence the focusing capability of the system is also reduced (It will be assessed in Section 4). Considering the description shown in Figure 5, the solution vector for this option is defined as:
  (14) $$\begin{array}{cc} \alpha_{MPP} & ={\left[\omega_{R}^{T},\omega_{I}^{T},x_{r,\omega }^{T},y_{c,\omega }^{T}\right]}^{T}\\ \alpha_{PP} & ={\left[∠\omega^{T},x_{r,\omega }^{T},y_{c,\omega }^{T}\right]}^{T} \end{array}$$
  where $x_{r,\omega }={\left[x_{r,\omega }\left[0\right],x_{r,\omega }\left[1\right],\ldots ,x_{r,\omega }\left[T_{x}-1\right]\right]}^{T},y_{c,\omega }={\left[y_{c,\omega }\left[0\right],y_{c,\omega }\left[1\right],\ldots ,y_{c,\omega }\left[T_{y}-1\right]\right]}^{T}$. Thus, the dimension of $\alpha_{MPP}$ and $\alpha_{PP}$ is $A=2T+T_{x}+T_{y}$ and $A=T+T_{x}+T_{y}$ respectively. The partial derivatives required in (9) are defined as the sum of the ones shown in (12) for each row or column:
  (15) $$\begin{array}{cc} \;\; & \;\;\frac{\partial E_{R}\left[n\right]}{\partial x_{r,\omega }\left[t_{x}\right]}\;=\;\sum_{t_{y}=0}^{T_{y}-1}\frac{\partial E_{R}\left[n\right]}{\partial x_{\omega }\left[t\right]}\\ \;\; & \;\;\frac{\partial E_{I}\left[n\right]}{\partial x_{r,\omega }\left[t_{x}\right]}\;=\;\sum_{t_{y}=0}^{T_{y}-1}\frac{\partial E_{I}\left[n\right]}{\partial x_{\omega }\left[t\right]}\\ \;\; & \;\;\frac{\partial E_{R}\left[n\right]}{\partial y_{c,\omega }\left[t_{y}\right]}\;=\;\sum_{t_{x}=0}^{T_{x}-1}\frac{\partial E_{R}\left[n\right]}{\partial y_{\omega }\left[t\right]}\\ \;\; & \;\;\frac{\partial E_{I}\left[n\right]}{\partial y_{c,\omega }\left[t_{y}\right]}\;=\;\sum_{t_{x}=0}^{T_{x}-1}\frac{\partial E_{I}\left[n\right]}{\partial y_{\omega }\left[t\right]} \end{array}$$
  where $t=\{t_{x},t_{y},t_{z}\}$.
- Elements follow certain model or mathematical function. The position of elements may be distributed according to a certain function, which is determined by a set of coefficients or hyperparameters. Generally, this set is smaller than the original number of positions, so the number of variables involved in the optimization is reduced. As an example, a paraboloid function may be used to model the array structure as:
  (16) $$z_{\omega }\left[t\right]=\frac{x_{\omega }{\left[t\right]}^{2}}{a^{2}}+\frac{y_{\omega }{\left[t\right]}^{2}}{b^{2}},\;\;\;t=0\ldots T-1$$
  Considering fixed $x_{\omega },y_{\omega }$, the optimization is limited to a,b, hence modifying $z_{\omega }$. Thus, only two parameters are used in the optimization, in order to modify the curve of the array (component $z_{\omega }$) instead of positions $\left\{x_{\omega },y_{\omega }\right\}$:
  (17) $$\begin{array}{cc} \alpha_{MPP} & ={\left[\omega_{R}^{T},\omega_{I}^{T},a,b\right]}^{T}\\ \alpha_{PP} & ={\left[∠\omega^{T},a,b\right]}^{T} \end{array}$$
  As a result, the optimization process is faster without degrading the array capabilities of focusing; in contrast, the fabrication tasks become more complex. An example is depicted in Figure 6. The partial derivatives are defined as:
  (18) $$\begin{array}{cc} \;\; & \;\;\frac{\partial E_{R}\left[n\right]}{\partial a}\;=\;\sum_{t=0}^{T-1}\frac{2\left(z_{\omega }\left[t\right]\;-\;z\left[n_{z}\right]\right)x_{\omega }{\left[t\right]}^{2}}{a^{3}R^{2}\left[n,t\right]}L_{1}\left[n,t\right]\\ \;\; & \;\;\frac{\partial E_{I}\left[n\right]}{\partial a}\;=\;\sum_{t=0}^{T-1}\frac{2\left(z_{\omega }\left[t\right]\;-\;z\left[n_{z}\right]\right)x_{\omega }{\left[t\right]}^{2}}{a^{3}R^{2}\left[n,t\right]}L_{2}\left[n,t\right]\\ \;\; & \;\;\frac{\partial E_{R}\left[n\right]}{\partial b}\;=\;\sum_{t=0}^{T-1}\frac{2\left(z_{\omega }\left[t\right]\;-\;z\left[n_{z}\right]\right)y_{\omega }{\left[t\right]}^{2}}{b^{3}R^{2}\left[n,t\right]}L_{1}\left[n,t\right]\\ \;\; & \;\;\frac{\partial E_{I}\left[n\right]}{\partial b}\;=\;\sum_{t=0}^{T-1}\frac{2\left(z_{\omega }\left[t\right]\;-\;z\left[n_{z}\right]\right)y_{\omega }{\left[t\right]}^{2}}{b^{3}R^{2}\left[n,t\right]}L_{2}\left[n,t\right] \end{array}$$

The choice of one of these structures may be conditioned by manufacturing issues. The free optimization of element locations typically lead to complicated structures whose implementation may be complicated. For that reason, depending on the characteristics of the final antenna to be implemented a proper choice might be the use of a non-uniform structure in rows and columns, also taking advantage of the increased number of degrees of freedom, but resulting in a quite simpler structure whose implementation is not much more complicated than taht of a uniform array. Obviously, a middle term may be found by using the proposed structure based on a predefined mathematical function, such as a paraboloid, or a horn. Additionally, the selection of different implementation technologies may have an impact of the bandwidth of the resulting antenna. In the case of applications such as Wireless Power Transfer such bandwidth may not be important, but in the case of applications involving information transfer, this is another element to account for when deciding the structure to be optimized and the set of constraints to be applied.

## 4. Results

NF-MF performance is analyzed in this Section, considering the effect of position optimization and the use of constraints for the magnitude and the phase of the feeding weights during the synthesis. The three mesh topologies presnted in Section 3.3 have been explores in order to perform the experiments. Two NF-MF antennas have been designed according to the scheme in Figure 1. For comparison purposes, the results presented in [7] are used as a reference. There, a 16 × 16 planar array with inter-element distance d=λ and located at the plane z=0 is intended to focus on $P_{1}=\left(P_{1,x},P_{1,y},P_{1,z}\right)=\left(2,0,9\right)\lambda$ and $P_{2}=\left(P_{2,x},P_{2,y},P_{2,z}\right)=\left(-4,0,12\right)\lambda$ simultaneously, using PP optimization. The same array has been used to focus on those targets using MPP and PP synthesis, as well as an 8 × 8 element planar array, also located at z=0. This second array has been chosen to be smaller in order to evaluate if the resultant reduced aperture size may be compensated by some increased degrees of freedom obtained optimizing more variables. Obviously, as focusing performance strongly depends on the size of the aperture and the geometry of the scenario [7,20], for a given problem (focal points) the 16 × 16 array should perform better than the 8 × 8 antenna, which is actually a subset of the bigger one. However, MPP optimization has been used with this 8 × 8 array in order to provide it with a greater number of degrees of freedom (due to the use of the magnitude as a free variable), comparing then the performance of both arrays with respect to the array tested in [7].

Table 1 and Table 2 show the results obtained after convergence of the optimization for the MPP and the PP optimization respectively. A dynamic μ has been used for the LM algorithm. The pair of template functions of low restrictiveness in $P_{1}$ and $P_{2}$ used in [7] to define the bounds $G_{M}\left[n\right],G_{m}\left[n\right],\forall n$ has been reproduced and specified. An original distance of $d_{x}=d_{y}=0.75\lambda$ and a minimum distance of $d_{yf}=d_{xf}=0.25\lambda$ were established for the two planar meshes; these values allow a range of 0.5λ for possible location of each element and a maximum and minimum distance among elements of 1.25λ and 0.25λ, respectively (punctual elements are considered for simplicity, but their size can be easily included in the method). On the other hand, |ω[t]|=1,∀t was considered for PP synthesis. Multifocusing performance was measured using the error rates defined in [7]: A mean error $\bar{F}$ (*F* in (5) normalized by *N*) and three complementary error rates to measure focusing accuracy ($F_{foc}$), width of the −3 dB focal spot ($F_{3dB}$), and achieved field level at the targets ($F_{\Delta P}$). Additionally, the distance between the defined focal points and the achieved maximal points $D_{FM}$ has been included, as well as the normalized field level at the focal point (where the field leve in the NF region is normalized to 1), denoted $|\bar{E}{|}^{2}$.

The obtained error rates show that the paraboloid-distribution of elements does not provide a real improvement with respect to the best tests without position synthesis (MP and PO in [7]) in the same number of iterations, but the position optimization improves the focusing capabilities for the planar meshes: better focusing, beamwidth and maximum levels, as well as lower distances between the designed focal point and the actual maximum synthesized. As the number of variables to optimize is increased, the temporal costs also increase, with an average of 31 s per iteration (on the before mentioned Intel Core i5-7500 PC with 3.4 GHz processor and 8 GB RAM) in the worst case (PP in 16 × 16 planar array with a general non-uniform lattice), while the results in [7] need 22 s per iteration for PO in the same array. The computation time required for each method is represented for the 16 × 16 element array in Table 3, in order to illustrate the differences between each optimization case. It is interesting to notice that the optimization of phases requires less iterations than the optimization of magnitudes and phases (due to the reduction in the number of variables to be optimized), but each iteration takes more time (due to the more complicated handling of phases for the algorithm; in the case of MP optimization, the weights are expressed in terms of their real and imaginary parts).

**Figure 7:**
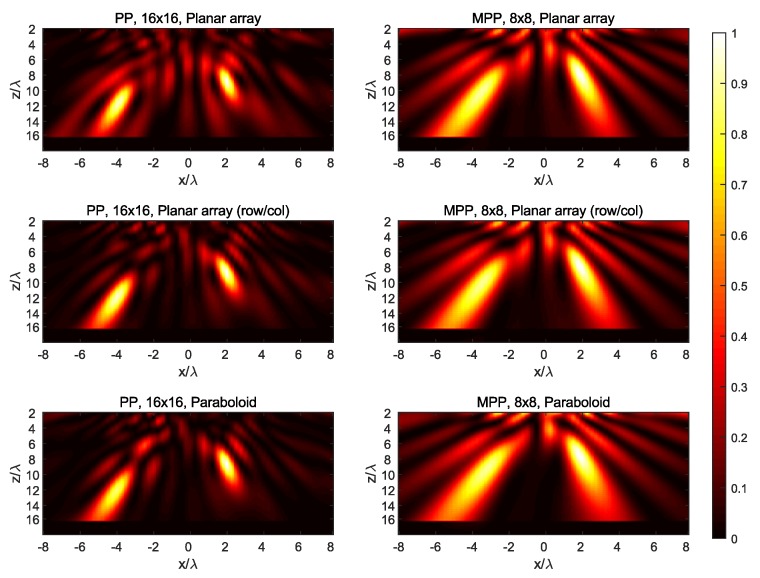
Normalized magnitude, ${\left(V/m\right)}^{2}$, of the synthesized radiated fields ${\bar{|\vec{E}\left[n\right]|}}^{2}$ for *n* corresponding to the plane $y=0=P_{1,y}=P_{2,y}$, where the targets are located.

**Figure 8:**
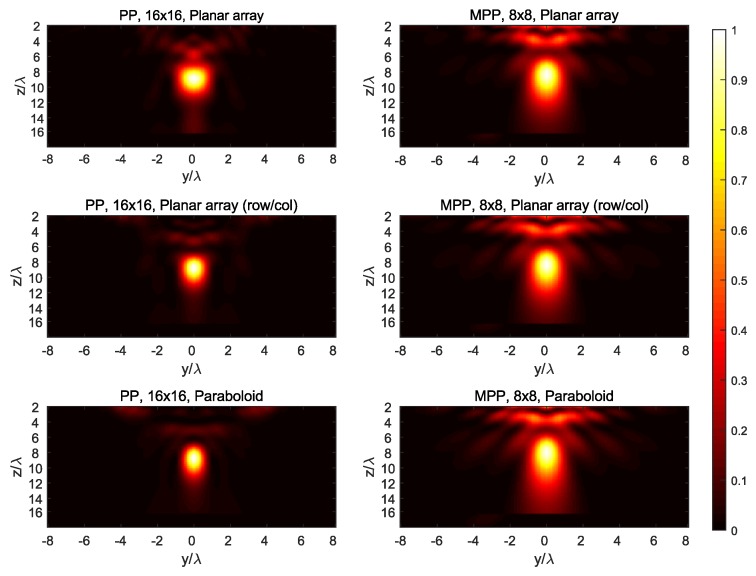
Normalized magnitude, ${\left(V/m\right)}^{2}$, of the synthesized radiated fields ${\bar{|\vec{E}\left[n\right]|}}^{2}$ for *n* corresponding to the plane $x=2=P_{1,x}$, where target #1 is located.

**Figure 9:**
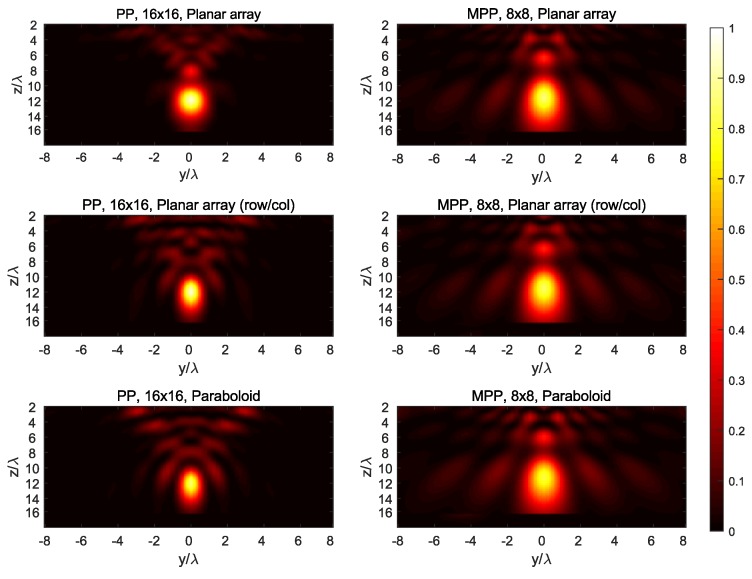
Normalized magnitude, ${\left(V/m\right)}^{2}$, of the synthesized radiated fields ${\bar{|\vec{E}\left[n\right]|}}^{2}$ for *n* corresponding to the plane $x=-4=P_{2,x}$, where target #2 is located.

**Figure 10:**
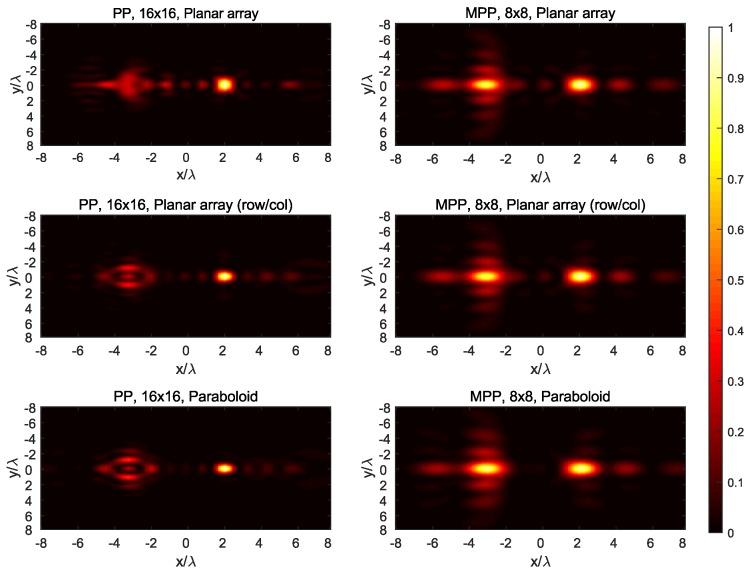
Normalized magnitude, ${\left(V/m\right)}^{2}$, of the synthesized radiated fields ${\bar{|\vec{E}\left[n\right]|}}^{2}$ for *n* corresponding to the plane $z=9=P_{1,z}$, where target #1 is located.

**Figure 11:**
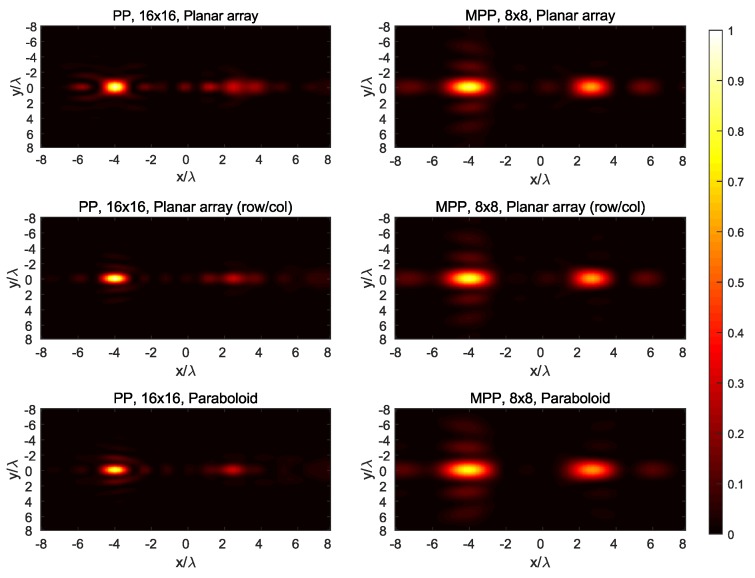
Normalized magnitude, ${\left(V/m\right)}^{2}$, of the synthesized radiated fields ${\bar{|\vec{E}\left[n\right]|}}^{2}$ for *n* corresponding to the plane $z=12=P_{2,z}$, where target #2 is located.

**Figure 12:**
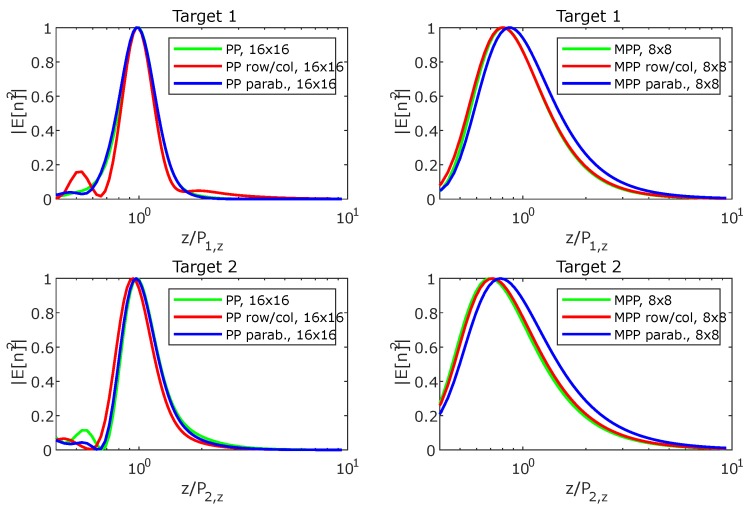
Normalized magnitude, ${\left(V/m\right)}^{2}$, of the synthesized radiated fields ${\bar{|\vec{E}\left[n\right]|}}^{2}$ along the *z*-axis passing through the focal points where the targets are located.

The synthesized field at the three main planes for both targets is shown in Figure 7, Figure 8, Figure 9, Figure 10 and Figure 11, while Figure 12 shows the normalized field along the *z*-axis passing through both focal points, where focusing performance may be noticed. The 8×8 array presents the worst performance, with the widest spot lobes, which demonstrates that the number of array elements (and therefore the size of the antenna) is the most relevant parameter for focusing performance.

**Figure 13:**
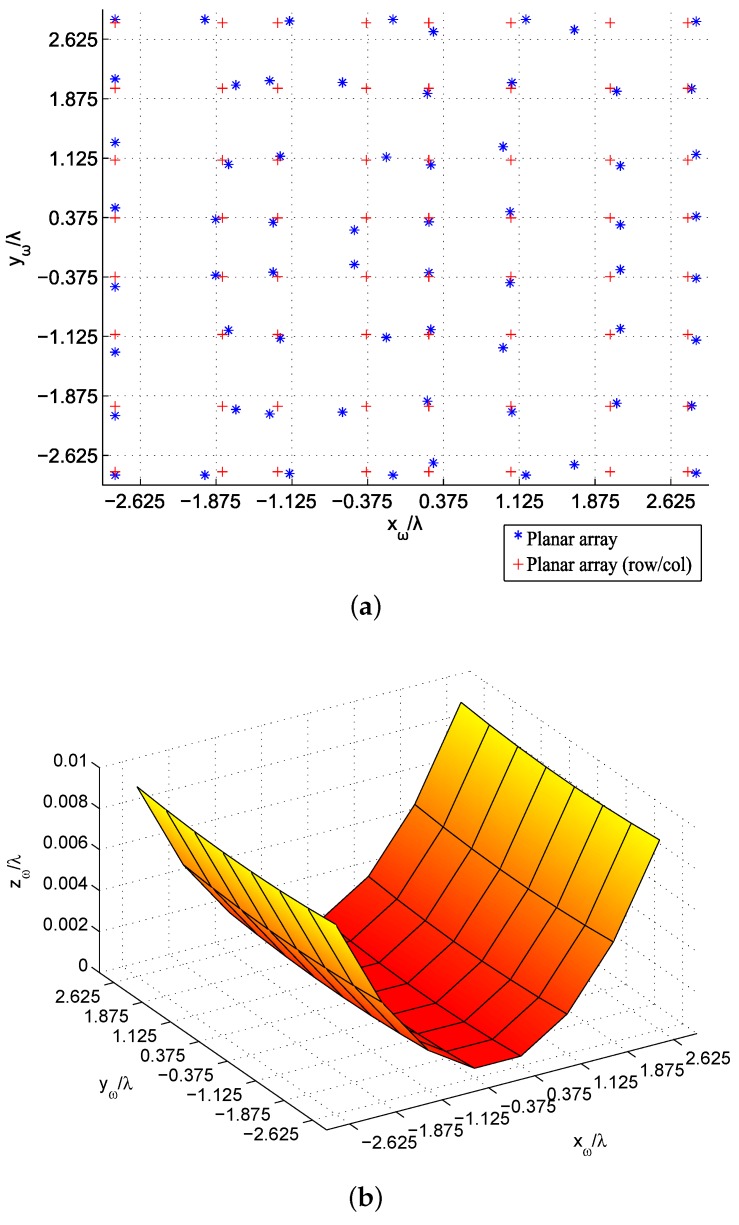
Obtained positions after the Magnitude-Phase-Position (MPP) synthesis of the 8 × 8 array in the studied cases: planar array (**a**) and paraboloid mesh (**b**).

**Figure 14:**
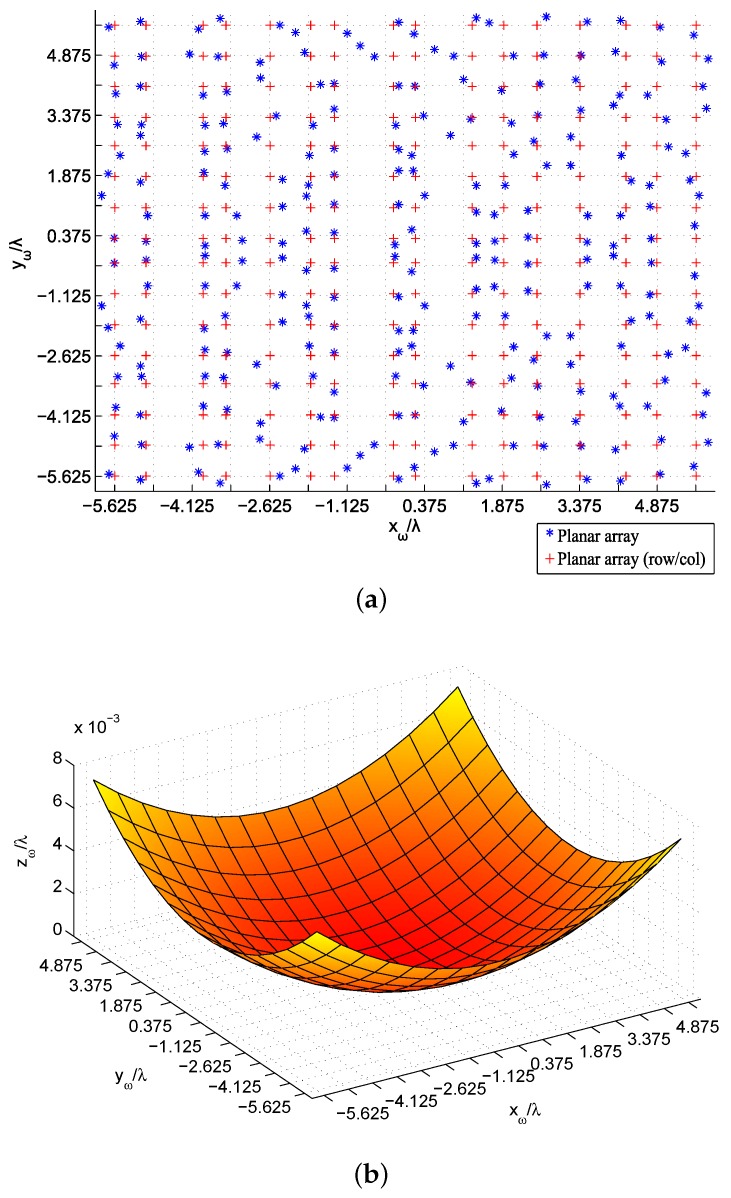
Obtained positions after the Phase-Position (PP) synthesis of the 16 × 16 array in the studied cases: planar array (**a**) and paraboloid mesh (**b**).

The resulting meshes obtained for the six tests are plotted in Figure 13 and Figure 14. The original mesh is depicted with dotted lines in Figure 13a and Figure 14a, and the final ones with crosses (for distributions in rows/columns) and asterisks (in case of a general non-uniform lattice). It is worth noting the symmetry in positions with respect to the plane y=0 in both cases, since this is the plane where the targets are located. As mentioned before, the dimension of the antenna is relevant for the focusing accuracy, so that the obtained locations for the elements in the 8 × 8 array intend to move away from the center of the array in order to increase the antenna surface and to deal with more demanding objectives. On the other hand, the 16 × 16 antenna is large enough so that that expansion is not necessary and the most significant locations are set in the center region of the array, related to the target planes. The expansion of the paraboloid functions (Figure 13b and Figure 14b) is also noted in their coefficients: the small array for MPP obtains {a=27.49,b=153.38}, which provide positions $z_{\omega }$ closer to the targets in order to fulfill the specifications. However, the 16 × 16 antenna obtained using PP presents {a=93.14,b=90.28}, leading to a more planar antenna array: in order to fulfill those requirements, such extension is not needed when an enough number of elements (enough size of the antenna) is provided.

Showing these results, a position optimization following a distribution in rows and columns could be an interesting trade-off among accuracy, temporal costs, and fabrication issues, since the results are similar to other complex meshes, such as the non-uniform planar lattice.

## 5. Conclusions

A synthesis framework for Near-Field Multifocusing has been extended to include optimization of the positions where the array elements must be located. The formulation of the cost function as a Least Squares problem allows the specification of a flexible set of variables to be obtained. This fact leads to a number of uniform and non-uniform structures to be possible when designing the array. The obtained lattices and feeding weights (real and imaginary parts in a MPP synthesis, or only phases for a PP optimization) are applied to the array in order to focus on several points simultaneously. Moreover, different Near-Field requirements may be achieved by modifying the specification of bounds in the provided cost function, finding solutions for more demanding problems since more variables are optimized.

Some examples demonstrate the flexibility of the proposed method, where different element distributions are achieved, including non-uniform planar arrays or even three-dimensional arrays where element locations follow certain predefined geometric functions. In these terms, the design of a planar array following uniform distribution in rows and columns is found to be a useful trade-off between NF performance and fabrication complexity. Although the temporal costs are increased compared to the synthesis of uniform arrays both using previously proposed MP or PO optimization, the error rates are reduced in terms of focusing performance, beamwidth, and maximum lobe levels. As the number of variables to optimize is higher, focusing capabilities (which always depend on the physical limitations of the antenna) are improved, thus justifying the use of position synthesis. However, the results show that the most relevant impact on focusing performance is still given by the size of the aperture, directly related to the number of elements in the array. Other degrees of freedom might be used to improve the results, but they do not compensate the advantages of a larger radiating system.

The experiments carried out have considered two focal spots. The proposed method does not limit the number of focal points to be defined, so any number of them may be considered. However, and related to the idea of focusing performance for a given aperture, a larger number of focal points means more demanding requirements, typically leading to a reduction in the focusing performance at each individual focal point. This effect is especially noticeable in the case of different focal lengths, with focal points located at positions with a difference of depth significant in relation with the aperture length. In such cases, a proper definition of the cost function may be used to weigth the importance of each focal point and to try to compensate their different focal lengths.

## Figures and Tables

**Table 1:** Error rates for Magnitude-Phase-Position (MPP) synthesis in an 8 × 8 array with initial $d_{x}=d_{y}=0.75\lambda$ (50 iterations, N=240,825, sampling each $\Delta \vec{r}\left[n\right]=0.25\lambda$, dynamic μ). T1 and T2 represent targets #1 and #2 respectively.

Mesh	$\;\bar{F}\;$	$\;F_{3\mathrm{dB}}\;$	$\;F_{\mathrm{foc}}\;$	$\;F_{\Delta P}\;$	$D_{\mathrm{FM}}^{T1}$	$|\bar{E}{|}_{T1}^{2}$	$D_{\mathrm{FM}}^{T2}$	$|\bar{E}{|}_{T2}^{2}$
Planar array	$2.50\;\times \;10^{-3}$	3.12λ	1.54	$9.70\;\times \;10^{-2}$	0.13λ	0.944	0.18λ	0.852
Planar array (col/row)	$2.71\;\times \;10^{-3}$	3.15λ	1.54	0.10	0.14λ	0.969	0.17λ	0.841
Paraboloid function	$4.81\;\times \;10^{-3}$	3.42λ	1.89	0.10	0.16λ	0.947	0.29λ	0.785
Best MP 8 × 8 uniform array in [7]	$3.21\;\times \;10^{-3}$	3.17λ	1.70	0.13	0.15λ	0.873	0.38λ	0.698

**Table 2:** Error rates for Phase-Position (PP) synthesis in a 16 × 16 array with initial $d_{x}=d_{y}=0.75\lambda$ (50 iterations, N=240,825, sampling each $\Delta \vec{r}\left[n\right]=0.25\lambda$, dynamic μ). T1 and T2 represent targets #1 and #2 respectively.

Mesh	$\;\bar{F}\;$	$\;F_{3\mathrm{dB}}\;$	$\;F_{\mathrm{foc}}\;$	$\;F_{\Delta P}\;$	$D_{\mathrm{FM}}^{T1}$	$|\bar{E}{|}_{T1}^{2}$	$D_{\mathrm{FM}}^{T2}$	$|\bar{E}{|}_{T2}^{2}$
Planar array	$8.36\;\times \;10^{-4}$	1.86λ	0.12	$1.51\;\times \;10^{-3}$	0	1	0.1λ	0.952
Planar array (col/row)	$9.82\;\times \;10^{-4}$	1.82λ	0.12	$1.70\;\times \;10^{-3}$	0	1	0.1λ	0.963
Paraboloid function	$6.98\;\times \;10^{-4}$	2.13λ	0.40	$5.90\;\times \;10^{-2}$	0	1	0.2λ	0.873
Best PO 16 × 16 uniform array in [7]	$1.20\;\times \;10^{-3}$	1.92λ	0.67	$5.05\;\times \;10^{-2}$	0.02λ	0.972	0.1λ	0.6981

**Table 3:** Computation time for different optimization cases in the 16×16 element array.

Optimization Case	Time per Iteration	Iterations
MP	18 s	45
PO	22 s	32
MPP	25 s	120
PP	31 s	80

## Abbreviations

The following abbreviations are used in this manuscript:

NF	Near-Field
NFF	Near-Field Focusing
CP	Conjugate-Phase
NF-MF	Near-Field Multifocusing
WPT	Wireless Power Transfer
WPIT	Wireless Power and Information Transfer
IoT	Internet of Things
LS	Least Squares
LM	Levenberg-Marquardt
MP	Magnitude-Phase
PO	Phase-Only
MPP	Magnitude-Phase-Position
PP	Phase-Position
